# Sexting Among Australian Adolescents: Risk and Protective Factors

**DOI:** 10.1007/s10964-023-01827-1

**Published:** 2023-07-22

**Authors:** Dominika Howard, Hannah K. Jarman, Elizabeth M. Clancy, Heidi M. Renner, Rachel Smith, Bosco Rowland, John W. Toumbourou, Matthew Fuller-Tyszkiewicz, Bianca Klettke

**Affiliations:** 1 Faculty of Health, School of Psychology, Deakin University, Geelong, VIC 3220 Australia; 2 Center for Social and Early Emotional Development, Deakin University, Burwood, VIC 3125 Australia; 3 Centre for Adolescent Health, Murdoch Children’s Research Institute, Parkville, VIC 3052 Australia; 4 Department of Paediatrics, University of Melbourne, Melbourne, VIC Australia; 5 Monash University, Eastern Health Clinical School & Monash Addiction Research Centre, Richmond, VIC Australia

**Keywords:** Sexting, Risk and protective factors, Adolescents, Social Development Model

## Abstract

Although consensual sending of sexts between adolescents is considered developmentally appropriate, it may also entail a range of negative consequences. Current sexting research lacks a comprehensive theoretical framework identifying a range of risk and protective factors underpinning adolescent consensual sending of sexts across individual, interpersonal, and distal levels. Further, there is a lack of systematic evaluation of how the importance of these factors may vary across adolescent age. This study investigated the utility of the Social Development Model to predict a range of risk and protective factors across individual, family, peer, school, and community-level factors. The sample included 1302 teenagers from Victoria, Australia (*M*_*age*_ = 14.54, *SD* = 1.14, 50.8% girls). Results indicated that 146 (11.7%) participants sent a sext (76 boys and 70 girls). Logistic regression analyses revealed that the Social Development Model accounted for 45.8% of variance in sexting, with greater likelihood of sending sexts being associated with older age, prior sexual activity, school sector, physical activity, lifetime substance use, greater depressive symptoms, sensation seeking, and perceived substance availability in the community. Multigroup analyses revealed that lifetime substance use was associated with a greater likelihood of sending sexts among younger teens. Among older adolescents, adaptive coping was associated with reduced engagement in sexting, while higher parental overcontrol and family conflict increased the odds of sending sexts. Overall, sexting is associated with a range of modifiable factors potentially amenable to intervention.

## Introduction

Consensual sending of sexts among adolescents is considered normative (Bianchi et al., 2017). However, this behavior may also entail adverse consequences including poor mental health, reputational damage, in-person and online victimization, and potentially even legal ramifications (Doyle et al., 2021; Krieger 2017a). As adolescents may not be well-equipped to deal with the psychological, social, and legal sequelae associated with sending image-based sexts, an understanding of the risk and protective factors linked to this behavior is needed to identify areas for sexting prevention and intervention measures. Current research on sexting lacks a theoretical model that would comprehensively examine a range of risk and protective factors associated with consensual sexting, and how the importance of these factors may vary across adolescent age. The present study will address these gaps by adopting the Social Development Model (Catalano & Hawkins, 1996) to examine the correlates of sending sexts across individual, family, peer, school, and community levels among younger and older Australian adolescents.

### Sexting Prevalence and Associated Outcomes

Sexting constitutes a common form of online sexual communication. The behavior can entail sending, receiving, and forwarding of sexual content (typically encompassing texts, images, or videos) via electronic means, and can range from consensual to non-consensual in nature (Barroso et al., 2023; Klettke et al., 2019). While consensual sexting is perceived to be a normative expression of young persons’ sexual repertoire (Levine, 2013) and a need for intimacy or validation (Bianchi et al., 2017), non-consensual sexting can be considered potentially harmful. The latter encompasses sexting behaviors performed under pressure, coercion, or threat, instances when a person is exposed to sexual material unwillingly/without their consent, or when their sexual image or text is distributed to the audiences beyond the intended recipients (Laird et al., 2021).

A recent meta-analysis revealed that 19.3% of young persons under the age of 18 reported sending sexts (via messages, images, or videos), while 14.5% forwarded a sext without consent (Mori et al., 2022). Sending sexts among adolescents was found to increase with the age of respondents and more recent time of publication/data collection, with no differences noted across gender (Molla-Esparza et al., 2020; Mori et al., 2022). A review examining the characteristics of consensual and non-consensual sexting among persons aged 10–24 years revealed a similar trend, in that participation in both forms of sexting increased with age but tended to be more prevalent among males (Barroso et al., 2023). These findings suggest that engagement in sexting among young people is likely to continue and potentially increase.

Research on sexting among young persons has revealed that the behavior can be problematic, even in instances when it is consensual. The consensual sending of sexts has been linked to increased symptoms of stress, depression, anxiety, reports of suicidal ideation, and suicide attempts among adolescents aged between 10 and 19 years (Doyle et al., 2021; Frankel et al., 2018), with the effects for anxiety and depression being more pronounced among younger respondents (Mori et al., 2019). Some adolescent girls reported greater body image self-consciousness as a result of sending sexts, getting in trouble with their teacher, and more frequent negative consequences when sexts were sent to multiple boys or men, or outside a romantic relationship (Bragard & Fisher, 2022). Young people depicted in sexts, especially girls, were subjected to harsh judgment, derogatory labeling, and suffered reputational damage (Krieger, 2017a). Further, sending image-based sexts among adolescents was significantly associated with higher odds of experiencing emotional and physical dating and relationship violence, in-person sexual assault, bullying, cyberbullying, sextortion, and grooming (Doyle et al., 2021). Sextortion pertains to a situation in which a person is blackmailed, threatened, or forced, by means of their sexually explicit material, to provide more sexual images, engage in sexual activity, or comply with other demands made by a perpetrator (Tamarit et al., 2021; Wolak et al., 2018). Grooming refers to building rapport and “befriending” a victim to exploit them sexually (Tamarit et al., 2021).

Legal consequences may also ensue. In some United States jurisdictions, even consensual sexting between young people under the age of 18 may result in legal charges and sex offender registration, as sexting may fall under the category of the possession and/or distribution of child pornography (Strasburger et al., 2019). Similarly, in Australia, an underage person who produces, possesses, or shares sexually explicit images of a minor may incur criminal charges under the Commonwealth laws (eSafety Commission, n.d.). A guilty verdict may result in a young person’s registration as a sex offender and a criminal record, thereby affecting their prospects of volunteering and working in places where children are present (eSafety Commissionaire, n.d.). Researchers emphasize the need to distinguish between consensual and non-consensual sexting behaviors, as legal ramifications in situations where two teenagers willingly exchange their sexual images for the purpose of flirting are developmentally appropriate, and hence should not be criminalized (Strasburger et al., 2019).

While the sending of sexts is not always associated with negative consequences, especially if the behavior is consensual and occurs in the context of a romantic relationship (Van Ouytsel et al., 2018), some researchers consider it inherently risky. This is because even consensual sexting can ultimately lead to the non-consensual forwarding of sexts or sexting due to pressure (Pedersen et al., 2023). To develop appropriate intervention and prevention measures addressing sexting behaviors among adolescents, a thorough understanding of the cross-section of risk and protective factors associated with sexting is needed. While risk factors increase one’s proclivity to engage in health-compromising behaviors, protective factors buffer against such actions and predict healthy development (Cahir et al., 2003).

### The Social Development Model

According to the Social Development Model (Catalano & Hawkins, 1996), an individual’s behavior, in addition to intrapersonal characteristics, is shaped by transactional (reciprocal) interactions with their environment and social units encompassing family, peers, school, and the wider community.

Child and adolescent behavior, whether prosocial or problematic, arises through a young person’s socialization processes with family, friends, community, and the characteristics of these social units (prosocial versus antisocial). The Social Development Model postulates that positive interactions with social units create bonds characterized by fostering 1) attachments to others; 2) commitments to the actions of a given social unit - family, school, community; and 3) beliefs in the value systems congruent with a given social unit. Bonding with prosocial others serves as a protective factor against engagement in problem behaviors. When opportunities for prosocial behaviors are limited, young people may bond with antisocial others or appraise antisocial behaviors as more enticing. In these instances, antisocial or problematic behaviors tend to arise.

The Social Development Model shares some similarities with Bronfenbrenner’s Socio-Ecological Model applied to sexting in prior literature (Hunter et al., 2021). Both models can be used to investigate a range of person-context factors to explain adolescent behavior. However, the Social Development Model offers a measurable framework to test a range of *risk* and *protective* factors underpinning potentially problematic behaviors, including sexting. The model also explains how certain aspects of one’s environment may determine youth’s engagement in prosocial versus problematic actions. That is, the Social Development Model focuses on strong social bonds as a mechanism through which problem adolescent behaviors could be prevented. Therefore, positive relationships between adolescents and their parents, peers, school, and the broader community may constitute protective factors against behaviors such as sexting and could be targeted in sexting prevention and intervention measures.

### The Social Development Model and Sexting

#### Intrapersonal factors

In line with the Social Development Model, research to date has identified several risk and protective factors associated with sending sexts among adolescents. On an individual, intrapersonal level, ethnic background (non-White) and sexual orientation (non-exclusively heterosexual) were associated with a greater likelihood of sending sexts (Klettke et al., 2014). Engagement in sexting was also related to higher levels of sensation seeking, impulsivity, and substance use (Cooper et al., 2016), (risky) sexual activity, multiple sexual partners, lack of contraception use (Kosenko et al., 2017; Mori et al., 2019), lower religiosity (Atwood et al., 2017), deviant behavior, and poorer mental health (Mori et al., 2019). Experiences of relational aggression, bullying, and cyberbullying have also been linked to sending sexts (Doyle et al., 2021). Personal qualities such as fairness and authenticity, on the other hand, were protective against sending sexts, suggesting that these character strengths constitute the basis for more measured technology use among young persons and hence should be targeted and enhanced in sexting prevention and intervention programs (Yépez-Tito et al., 2021). However, it is noteworthy that the aforementioned research encompasses samples of adolescents across a broad age range from 10 to 19 years. Therefore, whether the significance of these risk and protective factors varies across adolescent age requires further investigation.

#### Interpersonal factors

Research regarding parenting and family functioning among adolescents aged 12–20 years revealed several correlates significantly associated with sending sexts. Poorer attachment to parents and overly restrictive parenting practices have been identified as risk factors for greater willingness to send sexts (Atwood et al., 2017). Lower family cohesion reported by adolescents constituted a risk factor for sending partially naked photos or images to someone online (Baumgartner et al., 2012). Conversely, good family communication was a protective factor against engagement in sexting, including the number of people to whom sexts were sent and sending sexts to regulate one’s emotions (Bianchi et al., 2019). Greater parental love and support characterized by clear rules and interest in and encouragement of young persons’ future (Hunter et al., 2021), higher quality of parent-youth communication, parental knowledge, and monitoring of adolescent online and offline behaviors (Confalonieri et al., 2020) have been associated with the reduced engagement in sexting among adolescents.

Some research, however, has revealed that not all family variables are associated with sexting. Perceived parental attitudes towards sexting were not significantly related to young persons’ sending of sexts to a romantic partner or others (Van Ouystel et al., 2017). Parental supervision of youth’s online activities or mobile phones was not related to teens’ willingness to send sexts (Atwood et al., 2017) and sending of sexts (Campbell & Park, 2014). Some of these studies included samples of older adolescents, e.g., aged 15–21 years (Van Ouystel et al., 2017), suggesting that parental influence on teens’ sexting behaviors may reduce as the latter mature and individuate. However, this potential effect, especially in the context of Australian families, needs to be examined further.

#### Distal factors

Considering more distal factors, research examining peer, school, and community factors revealed that older teens (mean age > 16 years) were more likely to engage in sexting behaviors if their friends did as well (Maheux et al., 2019; Rice et al., 2018), and if their peers held favorable attitudes towards the engagement in sexting (Van Ouytsel et al., 2017). These findings are not surprising as the influence of peer group on adolescent behavior increases with age (Centers for Disease Control and Prevention, 2021). Further, students under the age of 16 with lower academic performance were more likely to send sexts (Barrense-Dias et al., 2022), while those who reported greater school connectedness were at a lower risk of sending sexts (Hunter et al., 2021). Adolescents aged 13–14 years from low-income families were less likely to send sexts relative to teens from high-income families (Kim et al., 2020). One study examined perceived social support (from adults and peers) in relation to sexting among adolescents aged 11–19 years and found no effect (Valido et al., 2020). However, this study focused on sexting perpetration, such as coercing someone into sexting or sending a sext to the recipient who did not consent to receive it. Overall, the importance of community variables and the potential interaction of age with peer, school, and community factors on the consensual sending of sexts is poorly understood.

## The Current Study

Research regarding consensual sexting behaviors is lacking a comprehensive theoretical model examining a range of potentially modifiable risk and protective factors associated with the consensual sending of sexts. It also encompasses samples of adolescents with broad age ranges, thereby lacking a systematic investigation of the significance of these factors across adolescent developmental stage. Engagement in any problem behavior, for example substance use, is often influenced by a variety of intrapersonal and environmental characteristics operating at various levels. Therefore, the current study adopted the Social Development Model to examine a range of factors that may account for young people’s participation in sending sexts. The current study’s aims were two-fold. First, this study examined the utility of the Social Development Model in identifying a range of individual, family, peer, school, and community risk and protective factors associated with consensual sending of sexts (Aim 1). Second, it explored whether the significance of these factors may vary across participants’ age (Aim 2). In line with the Social Development Model, it was hypothesized that protective factors across individual, family, peer, school, and community would be associated with a lower likelihood of sending consensual sexts, while risk factors across these individual and social units would increase the likelihood of sending consensual sexts. Considering Aim 2, the following question was explored: Does the relationship between the risk and protective factors across intrapersonal, interpersonal, and distal levels and consensual sending of sexts vary across adolescent age?

## Method

### Participants

Participants were drawn from the baseline data of a cluster randomized control trial evaluating the Communities That Care (CTC) intervention, which is aimed at reducing the number of young people under the age of 18 who engage in alcohol use and other antisocial behaviors (Rowland et al., 2018a). Communities that Care is an international intervention program, the purpose of which is to modify problem behaviors among adolescents in the United States, the Netherlands, and Australia (Jonkman et al., 2009; Toumbourou et al., 2019). Delivered in five stages (see www.communitiesthatcare.org.au/5-phases-ctc), the objective of the CTC approach is to establish strong partnerships among community stakeholders that will assist with implementing evidence-based programs addressing health problem behaviors among adolescents (substance use, violence, or school drop-out; Feinberg et al., 2010).

The current study included adolescents from schools in Victoria, Australia. In total, 1311 participants took part in the study at baseline. A small proportion of respondents (*n* = 9) did not report their gender and were excluded from the analyses, due to lack of power. The final sample consisted of 1302 participants, see Table 1 below.

**Table 1:** Demographic characteristics of study participants

Variable	Number	Percentage
Gender^a^		
Girls	662	50.8%
Boys	640	49.2%
Age	*M* = 14.54, *SD* = 1.14	
	Range 12–18 years	
Born in Australia	1176	90.3%
Aboriginal or Torres Strait Islanders	33	2.5%
School type		
Independent	86	6.6%
Government	548	42.1%
Catholic	668	51.3%
Engaged in sexual activity^b^		
Yes	65	5.0%
No	735	56.5%
Did not provide answer	502	38.6%
Maternal educational attainment		
University degree	545	44.7%
High school	331	27.1%
Did not complete high school	175	14.3%
“I don’t know”	169	13.9%
Paternal educational attainment		
University degree	419	34.3%
High school	285	23.4%
Did not complete high school	300	24.6%
“I don’t know”	216	17.7%
Maternal employment		
Working full-time	603	49.7%
Working part-time	403	33.2%
Not working	186	15.3%
Retired	13	1.1%
Mother/stepmother does not live with me	9	0.7%
Paternal employment		
Working full-time	959	79.3%
Working part-time	107	8.9%
Not working	53	4.4%
Retired	9	0.7%
Father/stepfather does not live with me	81	6.7%

^a^Gender was measured by the following question: “Which gender do you identify as” with options encompassing *male*, *female* and *other*

^b^Sexual activity was measured via a dichotomous *yes* vs *no* question: “Have you ever had sex?”

### Procedure

After obtaining ethics approval from the Deakin University Human Research Ethics Committee (2015-261), the Victorian Department of Education and Training (2016_002982), and Catholic education offices in the four dioceses in Victoria, a two-stage recruitment process was adopted. Initially, schools that participated in the Smart Generation randomized controlled trial (Rowland et al., 2018b) were approached, followed by additional schools within each community to ensure balance across school types (government, independent, and Catholic). All government and independent schools that agreed to take part in the study were co-educational. Out of the recruited Catholic schools, 54% were single sex. All students in years 8 and 10 at participating schools were invited to complete the survey. Signed informed consent from the school principal (or appointed representative) and parent or guardian was required prior to participation. Small incentives were offered to schools, teachers, and students to increase the return of parental consent forms, with incentives not being contingent on participation. For instance, schools were offered between $5 and $10 for the receipt of each parental consent form, with the funds being dedicated to new sports and educational equipment. Some of the teacher incentives included a funded lunch for the class. Students received either stress toys, USB drives, or slap bands for returning their parental consent form. Further, students provided their consent to participate in the research by completing the survey. The incentives varied across educational bodies, dependent on departmental approval, ethics condition according to the school type, and school agreement. Not all the schools took up the offers provided to them.

The student survey was delivered by trained research staff and teachers who were briefed on survey delivery in advance. The survey was conducted online across most schools, supported by paper surveys for schools with limited internet connectivity or access to technology. Participation was voluntary. Students were briefed prior to commencement, and the survey was delivered across a standard period (50–60 minutes) during class time. Year 8 students took ~40–55 minutes to complete the study, while Year 10 pupils completed it within 30–40 minutes. However, some schools utilized a double period to ensure all students had sufficient time to complete the survey. Students who were absent during delivery were provided with a paper survey to complete at a separate time under teacher supervision.

### Measures

#### Sexting behaviors

A sexting questionnaire was developed for the purpose of this study, with sexts being operationalized as “sexually explicit images” sent via mobile phone. The definition and structure of the questions were based on prior research on sexting behaviors (e.g., Clancy et al., 2019, 2020; Howard et al., 2020). For sending sexts, participants were asked whether they “have ever sent a nude (sexually explicit image) via their mobile phone”, with responses scored on an ordinal scale (1 = *never*, 2 = *yes, in the past 3 months*, 3 = *yes, in the past 6 months*, 4 = *yes, in the past year*, and 5 = *yes, more than a year ago*). The measure was then converted into a dichotomous scale, with 0 = *never having sent a sext* and 1 = *have sent a sext*.

Participants who ever sent a sext were asked an additional question about “the reasons for sending the nude”, with response options such as *to be sexy/initiate sexual activity, as a form of self-expression, to be flirtatious/fun, because someone pressured me to do so, as a joke/to be funny, because another person asked me to, bullying/harassment, because I was affected by drugs or alcohol, other*. Participants were allowed to select one or more motivations.

##### CTC measures

The measures were adopted from the Communities that Care Youth Survey (Glaser et al., 2005) examining several risk and protective factors at individual, family, peer, school, and community levels that may underpin a range of young persons’ problematic behaviors. The survey has demonstrated validity and reliability among adolescent respondents, with reliability overall averaging .76–.78 in prior studies (Bond et al., 2000; Toumbourou et al., 2019). After recoding relevant items, scale scores were calculated as a mean of all responses, with higher values indicating higher levels of risk or protection. Responses for the Short Mood and Feeling Questionnaire (SMFQ; Angold et al., 1996) were summed, with higher values reflecting more depressive symptoms. Table 2 lists all scales along with their means, standard deviations, and measures of internal consistency.

**Table 2:** Measures included in the main analyses

Variable’s Name	Definition	Scale sample items	*n*	*M*	*SD*	Cronbach’s *α*
Individual risk factors						
Depressive symptoms^a^	Low mood, feelings of loneliness, and not being good enough.	Thirteen items, e.g., In the past two weeks I felt miserable or unhappy. Responses 1-3: 1 = *not true*, 2 = *somewhat true*, 3 = *true*. Adapted from Short Mood and Feeling Questionnaire (SMFQ; Angold et al., 1996).	1276	8.00	7.21	.94
Lifetime substance use	Use of cigarettes, alcohol, drugs, and substances like glue or aerosol spray.	Five items, e.g., In your lifetime have you ever smoked cigarettes? Responses 0-1: 0 = *never*, 1 = *one or more times*.	1302	0.49	0.50	.62
Sensation seeking	Engagement in behaviors that were dangerous or ‘felt good’ despite the consequences.	Three items, e.g., How many times have you done crazy things, even if they are a little dangerous? Responses 1-6: 1 = *never*, 2 = *I’ve done it but not in the past year*, 3 = *less than once a month*, 4 = *about once a month*, 5 = *2 or 3 times a month*, 6 = *once a week or more*.	1290	2.18	1.19	.76
Antisocial behavior	Engagement in stealing, violence towards others, having been arrested or suspended from school.	Eight items, e.g., How many time in the past year (12 months) have you sold illegal drugs? Responses 0-1: 0 = *never*, 1 = *1 or more times*.	1302	0.18	0.38	.76
Bullying perpetration	Bullying another student through name-calling or teasing.	Three items, e.g. Have you taken part in bullying another student(s) at your school recently? Responses 1-4: 1 = *no*, 2 = *yes, less than once a week*, 3 = *yes, about once a week*, 4 = *yes, most days*.	1291	1.38	0.60	.66
Transitions and mobility	Frequency of moving homes or schools since kindergarten.	Four items, e.g., Have you changed schools (including changing from primary to secondary school) in the past year? Responses 1-4: 1 = *never*, 2 = *1 or 2 times*, 3 = *3 or 4 times*, 4 = *5 times or more*.	1302	1.70	0.53	.54
Individual protective factors						
Social competence	Interpersonal skills, ability to communicate one’s own feelings and recognize other people’s emotions.	Six items, e.g., How are you at letting friends know you like them by telling them or showing them? Responses 1-4: 1 = *very bad at this*, 2 = *poor at this*, 3 = *good at this*, 4 = *very good at this*. Adopted from Adolescent Interpersonal Competence Questionnaire (Buhrmester, 1990) and Self-Efficacy Questionnaire for Children (Muris, 2001).	1288	3.03	0.55	.78
Adaptive coping/Self-blame^a^	Adaptive stress and coping abilities: being overly critical of one-self, having the capacity to work out the problem on their own.	Four items, e.g., When I have a problem, I am good at working it out. Responses 1-4: 1 = *NO!*, 2 = *no*, 3 = *YES!*, 4 = *YES!*	1284	2.41	0.55	.63
Emotional control^a^	Ability to control difficult emotions and self-soothe when nervous.	Four items, e.g., I know how to relax when I feel tense. Responses 1-4: 1 = *NO!*, 2 = *no*, 3 = *YES!*, 4 = *YES!*	1284	2.77	0.66	.80
Physical activity	Number of days one took part in physical activity > 60 min over the last seven days and a normal week.	Two items, e.g., Over the past seven days, on how many days were you physically active for a total of at least 60 minutes per day? Responses 0-7: 0 = 0 days, 1 = *1*, 2 = *2*, 3 = *3*, 4 = *4*, 5 = *5*, 6 = *6*, 7 = *7*.	1249	3.91	2.10	.93
Belief in moral order^a^	Importance of honesty, attitudes towards behaviors such as cheating at school or beating someone up if they start a fight first.	Four items, e.g., It is important to be honest with your parents, even if they become upset or you get punished. Responses 1- 4: 1 = *NO!*, 2 = *no*, 3 = *YES!*, 4 = *YES!*	1290	1.69	0.56	.68
Religiosity^a^	Importance of religion and frequency of engagement in religious activities.	Two items, e.g., How important is religion or spirituality in your life? Responses 1-5: 1 = *not important at all*, 2 = *not very important*, 3 = *somewhat important*, 4 = *very important*, 5 = *extremely important*.	1299	1.85	0.77	.76
Family risk factors						
Parental overcontrol	Perceptions of parents as being overly controlling.	Two items, e.g., My parents try to control everything I do. Responses 1-4: 1 = *NO!*, 2 = *no*, 3 = *YES!*, 4 = *YES!*	1189	2.25	0.85	.76
Poor family management	Perceptions of family rules and parental awareness of problematic behaviors like skipping school or drinking alcohol.	Nine items, e.g., My family has clear rules about alcohol and drug use. Responses 1- 4: 1 = *YES!*, 2 = *YES!*, 3 = *no*, 4 = *NO!*	1217	1.65	0.52	.83
Family conflict	Frequent arguments between family members, yelling and insults.	Three items, e.g., We argue about the same things in my family over and over. Responses 1- 4: 1 = *NO!*, 2 = *no*, 3 = *YES!*, 4 = *YES!*	1170	2.87	0.86	.84
Family history of antisocial behavior^a^	Drug and alcohol use among siblings and family members, engagement in behaviors such stealing or assaults.	Ten items, e.g., About how many adults over 18 have you known personally who in the past year have sold or delt drugs? Responses 1-4: 1= *none*, 2 = *1*, 3 = *2*, 4 = *3 or more*.	1200	1.74	0.78	.80
Family protective factors						
Family attachment^a^	Closeness with parents, sharing of thoughts and feelings with mother and father.	Four items, e.g., Do you feel close to your mother? Responses 1-4: 1 = *NO!*, 2 = *no*, 3 = *YES!*, 4 = *YES!*	1210	3.14	0.67	.79
Family opportunities for prosocial involvement^a^	Opportunities for doing fun things with parents, being able to rely on them and being included in the decision-making processes.	Three items, e.g., If I had a personal problem, I could ask my mum and dad for help. Responses 1-4: 1 = *NO!*, 2 = *no*, 3 = *YES!*, 4 = *YES!*	1215	3.12	0.69	.72
Family rewards for prosocial involvement^a^	Being rewarded for doing a good job, enjoying time spent with mother/father.	Four items, e.g., My parents notice when I am doing a good job and let me know about it. Responses 1- 4: 1 = *never or almost never*, 2 = *sometimes*, 3 = *often*, 4 = *all of the time*.	1220	3.18	0.63	.76
Peer risk factors						
Interaction with antisocial peers	Number of peers a young person is best friends with who stole something, were suspended from school, sold drugs or attacked someone.	Eight items, e.g., In the past year (12 months), how many of your four best friends have stolen something worth more than $10? Responses 1-5: 1 = *none of my friends*, 2 = *1 of my friends*, 3 = *2 of my friends*, 4 = *3 of my friends*, 5 = *4 of my friends*.	1292	1.30	0.59	.85
Peer protective factors						
Interaction with prosocial peers	Number of peers a young person is best friends with who do well at school, are members of sports or other clubs.	Two items, e.g., In the past year (12 months), how many of your four best friends have tried to do well at school? Responses 1-5: 1 = *none of my friends*, 2 = *1 of my friends*, 3 = *2 of my friends*, 4 = *3 of my friends*, 5 = *4 of my friends*.	1289	4.19	0.90	.45
School risk factors						
Academic failure	Evaluation of one’s own marks and in comparison to most other students in one’s class.	Two items, i.e., Putting them all together, what were your marks like last year? Responses 1-4: 1 = *very good*, 2 = *good*, 3 = *average*, 4 = *poor*.	1300	1.99	0.64	.70
Low commitment to school	Interest in and importance of school subjects, enjoyment of school, and effort put into schoolwork.	Seven items, i.e. During the last 4 weeks, when school was in session, how many whole days have you missed because you skipped or wagged? Responses 1-5: 1 = *none*, 2 = *1*, 3 = *2*, 4 = *3*, 5 = *4 or more days*.	1301	2.30	0.67	.82
School protective factors						
Opportunities for prosocial involvement at school^a^	Opportunities to get involved in special school activities, class discussions and decision-making processes pertaining to class activities and rules.	Five items, e.g., There are lots of chances for students in my school to get involved in sports, clubs, organizations or other school activities outside of class. Responses 1-4: 1 = *NO!*, 2 = *no*, 3 = *yes*, 4 = *YES!*	1295	2.85	0.57	.63
Rewards for prosocial involvement at school^a^	Feeling safe at school, receiving positive feedback from teachers.	Four items, e.g., My teachers notice when I am doing a good job and let me know about it. Responses 1-4: 1 = *NO!*, 2 = *no*, 3 = *YES!*, 4 = *YES!*	1294	2.85	0.57	.72
Community risk factors						
Community disorganization^a^	Crime, fights, drug-selling, presence of abandoned buildings in the neighborhood.	Five items, e.g. How much do each of the following statements describe your neighborhood – lots of graffiti. Responses 1-4: 1 = *NO!*, 2 = *no*, 3 = *YES!*, 4 = *YES!*	1185	1.56	0.58	.81
Low attachment to neighborhood^a^	Feelings towards one’s neighborhood and one’s readiness to move out of it.	Three items, e.g., I'd like to get out of my neighborhood. Responses 1-4: 1 = *NO!*, 2 = *no*, 3 = *YES!*, 4 = *YES!*	1159	1.86	0.71	.74
Perceived substance availability^a^	Ease of access to alcohol, cigarettes, or drugs in one’s neighborhood.	Four items, e.g., If you wanted to get some cigarettes, how easy would it be for you to get some? Responses 1–4: 1 = *very hard*, 2 = *sort of hard*, 3 = *sort of easy*, 4 = *very easy*.	1151	1.95	0.89	.85
Laws and norms favorable to drug use^a^	Perceptions of potential consequences for engaging in alcohol or drug use, and how most adults perceive adolescent substance use.	Six items, e.g., If a kid drank some alcohol (like beer, wine or spirits) in your neighborhood, would he or she be caught by the police? Responses 1-4: 1 = *YES!*, 2 = *YES!*, 3 = *no*, 4 = *NO!*	1165	2.05	0.62	.78
Community protective factors						
Opportunities for prosocial involvement in the community	Availability of sports clubs, youth groups, community services for teens, and supportive adults.	Seven items, e.g., In my neighborhood, adults pay attention to what kids have to say. Responses 1-4: 1 = *NO!*, 2 = *no*, 3 = *YES!*, 4 = *YES!*	1046	2.93	0.67	.73
Rewards for prosocial involvement in the community	Presence of supportive adults who encourage and praise effort made by teens.	Three items, e.g., There are people in my neighborhood who encourage me to do my best. Responses 1-4: 1 = *NO!*, 2 = *no*, 3 = *YES!*, 4 = *YES!*	1159	2.16	0.86	.91

^a^Relevant scale items were reverse-coded

Means and standard deviations based on available data; Reliability of two-item scales are reported using Spearman-Brown coefficients

### Analytic Approach

There was a small to moderate amount of missing data across all variables (0–13%), except for previous sexual intercourse, parental education, and opportunities within the community which had 38.6%, 25.6%, and 19.7% missing data, respectively. Based on Little’s MCAR test, data were not missing completely at random (*p* < 0.001). T-tests and chi-square tests were performed to determine whether study variables could differentiate individuals with and without missing data. Inspection of patterns of missing data indicated that individuals with missing data were more likely to be male and to report significantly higher religiosity and attachment to neighborhood, but score lower on depressive symptoms, transitions and mobility, family attachment, laws and norms favorable to drug use than individuals with no missing data. To handle missing data, multiple imputation with chained equations and dummy variables, to reflect clustering by school, was performed on 50 datasets (Lee & Carlin, 2010; White et al., 2010). The predictors of missingness identified above were included in the imputation model to correct for their potential biasing effects. However, sometimes key variables responsible for missingness are not detected by these simple tests. As a further approach, and consistent with best practice, a wide range of predictors were included in the imputation model to make the missing at random assumption more plausible (e.g., Madley-Dowd et al., 2019). Specifically, demographic measures, all time 1 and time 2 data (the latter of which is not analyzed in the present study) were included in the imputation model. For the main analyses, results were pooled across the 50 imputed datasets using Rubin’s (1987) rules.

Descriptive statistics were used to summarize the non-imputed data. Categorical variables were described using frequencies, while for continuous measures means and standard deviations were calculated (see Tables 1–3). To investigate associations between categorical variables, chi-square analyses were performed. To examine associations between continuous and categorical variables, bivariate correlations were conducted.

**Table 3:** Frequency of sending sexts based on gender and demographic variables

Variable	Ever Sent a Sext		χ²	*φ*
	Yes (n %)	No (n %)		
Boys	76 (12.4%)	536 (87.6%)	0.67	−.02
Girls	70 (10.9%)	570 (89.1%)		
Had sex	33 (54.1%)	28 (45.9%)	107.39***	.37***
Never had sex	62 (8.7%)	650 (91.3%)		
Independent school	12 (14.6%)	70 (85.4%)	5.43	.07
Government school	446 (86.3%)	71 (13.7%)		
Catholic school	63 (9.6%)	590 (90.4%)		

Fifty participants did not provide responses to the sexting question

***p < .001

For the main analyses, a logistic regression was conducted in Mplus version 8.7 (Muthén & Muthén, 2017) to examine cross-sectional predictors of sexting. The data were clustered by school, which can inflate standard errors. Therefore, to account for this, maximum likelihood-based robust standard errors (Huber-White sandwich estimator method) were used. Age, sex (gender), parental employment, parental education, school sector, and previous sexual intercourse were controlled for by being entered as additional predictors. Continuous predictors (e.g., age, bullying, physical activity) were entered into the model group-mean centered.

Multigroup analyses were conducted to test whether the study effects differed by age. Following the developmental cut-offs defined by the Centers for Disease Control and Prevention (2021), two groups were compared: young teens (12–14 years old; *n* = 667) and adolescents (15–17 years old; *n* = 625). Participants who were 18 years old or did not report their age were excluded from these analyses (*n* = 10). Two models were compared: one in which parameters were free to vary across age groups and one in which parameters were constrained to be equal across the groups. Equivalence of the model across age groups was then evaluated using Wald tests, which assess whether the strength of each effect differed significantly between groups.

## Results

### Descriptive Statistics and Preliminary Analyses

Of all respondents, 146 (11.7%) had ever sent a sext (76 boys, 70 girls), while 1106 (88.3%) had never engaged in this behavior. Boys and girls sent sexts to a similar degree, with no statistical difference based on gender χ²(1) = 0.67, *p* = .42, *φ* = −.02. Further, there was no relationship between having sent a sext and the type of school students attended *χ²*(2) = 5.43, *p* = .07, *φ* = .07. However, as illustrated in Table 3, there was a significant association between having sent a sext and engaging in sexual activity *χ²*(1) = 107.39, *p* < .001, *φ* = .37, with participants who reported having engaged in sexual activity being more likely to have ever sent a text.

Sexting motivations are reported in Table 4. The most frequent motivations for sending sexts were “to be sexy” or “flirtatious”. Seventeen (7.3%) respondents noted that they sent a sext “due to pressure”. However, further inspection of this category revealed that even among those participants, the majority (*n* = 10) simultaneously noted motivations such as “flirting”, “self-expression”, or “because someone asked them to”, suggesting that consent is not always categorical but may lie on a continuum.

**Table 4:** Summary of sexting motivations among adolescents who sent a sext

Motivation	*n*	%
To be sexy/initiate sexual activity	62	26.6
As a form of self-expression	12	5.2
To be flirtatious	61	26.1
Because someone pressured me	17	7.3
As a joke, to be funny	29	12.4
Because someone asked me	40	17.2
Bullying/harassment	0	0
Because I was affected by drugs or alcohol	5	2.1
Other reasons	7	3.0
Total	233	100

### Main Analyses

Table 5 presents the results of the multivariate model. Sexting was significantly associated with higher age, previous sexual intercourse, physical activity, lifetime substance use, sensation seeking, depressive symptoms, and perceived substance availability in the community. In addition, sexting was significantly related to the school sector, whereby higher levels of sexting were reported in independent and government schools compared to Catholic schools. Overall, the predictors accounted for 45.8% of the variance in sexting (*R*^*2*^ = .458, *p* < .001). According to Ferguson’s (2009) guidelines, this result constitutes a moderate-to-strong effect.

**Table 5:** Summary statistics for predictor variables on sending sexts

Demographic variables	*Β*	*OR*	*p*	95% CIs
**Age**	**0.201**	1.223	.034*	**[0.015, 0.388]**
Gender^a^	−0.090	0.914	.632	[−0.461, 0.280]
**Previous sexual intercourse**^**b**^	**1.369**	3.931	<.001***	**[0.647, 2.091]**
Parental employment status	−0.003	0.997	.986	[−0.382, 0.376]
Parental education	−0.171	0.843	.446	[−0.612, 0.269]
**School sector** **(independent vs catholic)**^**c**^	**0.909**	2.482	<.001***	**[0.553, 1.265]**
**School sector** **(government vs catholic)**^**d**^	**0.548**	1.730	.024*	**[0.071, 1.026]**
Individual protective factors				
**Physical activity**	**0.147**	1.158	.006**	**[0.041, 0.253]**
Belief in moral order	0.298	1.347	.066	[−0.020, 0.616]
Emotional control	−0.169	0.845	.311	[−0.495, 0.158]
Religiosity	0.047	1.048	.741	[−0.229, 0.323]
Social competence	−0.003	0.997	.989	[−0.488, 0.495]
Adaptive coping/Self-blame	−0.104	0.901	.674	[−0.591, 0.383]
Individual risk factors				
**Lifetime substance use**^**e**^	**1.061**	2.880	<.001***	**[0.578, 1.543]**
**Depressive symptoms**	**0.062**	1.064	.001**	**[0.026, 0.097]**
**Sensation seeking**	**0.287**	1.332	.016*	**[0.054, 0.520]**
Bullying	0.246	1.279	.253	[−0.176, 0.668]
Transitions and mobility	0.214	1.239	.235	[−0.139, 0.567]
Antisocial behavior^f^	−0.129	0.879	.636	[−0.661, 0.403]
Family protective factors				
Family opportunities for prosocial involvement	−0.129	0.879	.467	[−0.578, 0.265]
Family rewards for prosocial involvement	0.313	1.368	.199	[−0.164, 0.789]
Family attachment	−0.017	0.983	.945	[−0.511, 0.466]
Family risk factors				
Parental overcontrol	0.110	1.116	.545	[−0.283, 0.462]
Poor family management	−0.098	0.907	.738	[−0.669, 0.474]
Family history of antisocial behavior	0.114	1.121	.600	[−0.312, 0.540]
Family conflict	0.171	1.186	.357	[−0.192, 0.534]
Peer protective factors				
Interaction with prosocial peers	0.006	1.006	.976	[−0.390, 0.402]
Peer risk factors				
Interaction with antisocial peers	0.048	1.049	.802	[−0.326, 0.422]
School protective factors				
Opportunities for prosocial involvement at school	0.314	1.369	.302	[−0.282, 0.910]
Rewards for prosocial involvement at school	0.268	1.307	.362	[−0.308, 0.844]
School risk factors				
Academic failure	0.299	1.349	.236	[−0.195, 0.792]
Low commitment to school	−0.038	0.963	.873	[−0.503, 0.427]
Community protective factors				
Opportunities for prosocial involvement in the community	0.275	1.317	.192	[−0.138, 0.688]
Rewards for prosocial involvement in the community	−0.044	0.957	.761	[−0.329, 0.241]
Community risk factors				
**Perceived substance availability**	**0.535**	1.707	<.001***	**[0.265, 0.806]**
Laws and norms favorable to drug use	0.322	1.380	.100	[−0.061, 0.704]
Community disorganization	−0.116	0.890	.497	[−0.449, 0.218]
Low attachment to neighborhood	−0.098	0.907	.603	[−0.467, 0.271]

*p < .05, **p < .01, ***p < .001

Unstandardized coefficients are reported

Bold text indicates significant findings

*OR* odds ratio

^a^0 = male, 1 = female

^b^0=no/never, 1=yes

^c^1 = Independent, and Catholic school was the reference category

^d^1 = Government, and Catholic school was the reference category

^e^0 = no/never, 1 = yes

^f^0 = no/never, 1 = yes

The multigroup analyses revealed differences between young teens (aged 12–14 years) and older adolescents (aged 15–17 years) on four predictors, namely lifetime substance use (*B* = 1.453, 95% CI [0.165, 2.740]), adaptive coping (*B* = 1.239, 95% CI [0.094, 2.383]), parental overcontrol (*B* = −0.860, 95% CI [−1.652, −0.068]), and family conflict (*B* = −0.800, 95% CI [−0.491, −0.110]). Specifically, a positive association was found between sexting and lifetime substance use for young teens (*B* = 1.890, 95% CI [0.892, 2.888]) but not older adolescents (*B* = 0.437, 95% CI [−0.217, 1.091]). Conversely, sexting was negatively associated with adaptive coping for older adolescents (*B* = −0.661, 95% CI [−1.249, −0.073]) but not young teens (*B* = 0.577, 95% CI [−0.340,1.495]). Sexting was also positively associated with parental overcontrol (*B* = 0.482, 95% CI [0.028,0.936]) and family conflict (*B* = 0.596, 95% CI [0.155, 1.038]) in older adolescents but not young teens, respectively (*B* = −0.378, 95% CI [−1.009, 0.252]; *B* = −0.204, 95% CI [−0.764, 0.355]). The differences between young teens and adolescents on all other predictors were non-significant and can be obtained from the authors on request.

## Discussion

Although the consensual sending of sexts is often considered developmentally appropriate among adolescents (Bianchi et al., 2017), it can be associated with mental health and social problems (Doyle et al., 2021), and even legal repercussions (Strasburger et al., 2019). Current research on sexting among young persons is lacking a comprehensive framework that would identify a range of risk and protective factors associated with the consensual sending of sexts across individual, family, peer, school, and community factors. It is also missing an evaluation of how the significance of these factors may vary across adolescent developmental stages. This study applied the Social Development Model (Catalano & Hawkins, 1996) to identify sexting risk and protective factors among adolescents across individual and social units such as family, peer, school, and community. Overall, the risk and protective factors accounted for 45.8% of the variance in sending consensual sexts. These findings suggest that the Social Development Model constitutes a suitable framework for identifying a range of risk and protective factors associated with adolescent sexting.

The hypothesis predicting that protective factors across individual, family, peer, school, and community would be associated with a lower likelihood of sending consensual sexts, while risk factors across these individual and social units would increase the likelihood of sending consensual sexts, was partially supported. In contrast to what was anticipated, higher scores on physical activity (an individual protective factor) were associated with the increased odds of ever having sent a consensual sext. In prior research, physical activity has been identified as a protective factor among adolescents against problematic behaviors, such as substance use (Simonton et al., 2018; Thompson et al., 2020). It is important to note that the current study explored the consensual sending of sexts frequently endorsed by motivations such as initiating sexual activity and flirting. It is possible that physical activity underpins a facet of sexting behavior that, in the eyes of adolescents, is considered common and constitutes a part of one’s dating strategy. Specifically, prior research revealed that adolescents often sent sexts to initiate sexual activities, increase passion within a romantic relationship, and verify whether they are perceived as attractive by their peers (Bianchi et al., 2017). A study conducted among 18–21-year-olds found that positive evaluations of one’s body were correlated with a greater likelihood of sending sexts (Howard et al., 2021). Physical activity may therefore constitute a mediating factor between body image satisfaction and the consensual sending of sexts. ‘Good looks’ tend to be valued among teenagers (Ringrose et al., 2013; Ringrose & Harvey, 2015), with those perceived as athletic being appraised as more attractive by their peers (Vannatta et al., 2009). As such, physical activity and athletic physique may embolden young people to send sexts to explore romantic interests and seek validation from their peers, with both motivations constituting developmental goals for this age (Bianchi et al., 2017).

In line with the predicted direction, higher scores on lifetime substance use, depressive symptoms, sensation seeking (individual risk factors), and perceived substance availability in the community (community risk factor) were associated with the increased likelihood of sending sexts among adolescents. These results corroborate prior research whereby adolescents who engaged in substance use scored higher on sensation seeking (Cooper et al., 2016) and depressive symptoms (Doyle et al., 2021; Frankel et al., 2018) were more likely to engage in the consensual sending of sexts. The current study extends these findings by illustrating that a more distal factor relating to substance use, such as perceived substance availability in the community (a community risk factor), was also positively associated with higher odds of sexting. It is possible that greater access to substances may be associated with young persons’ perceptions of their communities as more permissive towards problematic behaviors, like alcohol use. These perceptions may also disinhibit young people from engaging in other potentially risky behaviors, including sexting. Further, perceived substance availability may also be linked to the actual consumption of drugs and alcohol among teens, which was found to increase the odds of engaging in sexting in this study and prior research (Frankel et al., 2018).

The present study also explored whether the range of individual, family, peer, school, and community risk and protective factors may vary in significance across adolescent age. Lifetime substance use emerged as an individual risk factor among younger teens (12–14 years) but not among older adolescents (15–17 years). Among the latter, increased scores on adaptive coping (individual protective factor) predicted lower engagement in sending consensual sexts. Yet, higher scores on family risk factors such as parental overcontrol and family conflict increased the odds of ever having sent a consensual sext. Prior research has shown that substance use constitutes a risk factor for sending sexts that increases with age (Mori et al., 2019). This study revealed that in Australia, substance use is a significant risk for sexting among *younger* teens, suggesting that the age between 12–14 years constitutes an important period for interventions addressing a range of risky behaviors such as substance use, sexting, and associated sexual activity (Kosenko et al., 2017).

This study also found that the prominence of family variables, such as family conflict and parental overcontrol, emerged among older adolescents, which contrasts with some of the studies conducted among older teens and young adults in the past (Van Ouystel et al., 2017). Teens aged 15–17 years show more interest in developing romantic relationships with their peers and are more likely to spend less time with their parents (Centers for Disease Control and Prevention, 2021). As such, when parents are perceived as overly controlling or intrusive, and family conflict is rife, young people might be potentially rebelling against parental overcontrol and seeking affiliation with peers through the engagement in the consensual sending of sexts.

It is noteworthy that only one factor was identified as protective against sending sexts among adolescents aged 15–17 years. Adaptive coping, operationalized as an ability to work through a problem on one’s own and being less self-critical, was associated with lower odds of sending sexts. This is an important finding as adaptive coping may be underpinning young persons’ sexting self-efficacy and the ability to exert influence over their sexting behaviors. In a study by Howard et al. (2022), albeit conducted among emerging adults aged 18–25 years, lower levels of self-efficacy were associated with sending consensual and non-consensual sexts. In the present study, higher scores on adaptive coping were associated with lower odds of sexting potentially through a young person’s ability to refuse to sext if external pressure was applied or internal pressure was experienced.

### Implications

The present study illustrated that the Social Development Model (Catalano & Hawkins, 1996) proved to be an optimal framework for identifying a range of risk and protective factors across individual and social units, explaining 45.8% of the variance in consensual sending of sexts. This study revealed that to understand adolescent sexting, a sound theoretical framework examining a range of factors across intra- and interpersonal levels is needed. Further, researchers should consider adolescent age to ascertain which factors and developmental stages predispose young persons to engage in sending sexts.

Regarding practical implications, the current study illustrated that in addition to individual factors such as substance use, depressive symptoms, or sensation seeking, sexting prevention and intervention measures could maximize their impact by simultaneously addressing more distal factors such as the perceived availability of substances in the community. Further, this study revealed that parents and educators should monitor substance use, especially among younger teens aged 12–14 years. This is because substance use at this age may also constitute a marker for other problematic behaviors, such as engagement in sext sending. Conversely, parents of adolescents aged 15 to 17 years should be made aware that restrictive parenting and chaotic family environment may ‘backfire’ among older teens who, through sexting, may be rebelling against parental control and be more willing to seek comfort, validation, and understanding from romantic partners. As such, parents could potentially seek advice from trained professionals on how to adjust their parental style to better align with the developmental needs of older teens. In line with the findings of this study, strategies could encompass supporting young persons’ autonomy by reinforcing their ability to work through challenges, establishing better communication with teens, and improving family functioning. These approaches could potentially prevent sexting among adolescents and the associated problematic offline sexual behaviors such as sex with multiple partners or sex without contraception (Mori et al., 2019).

### Study Limitations and Directions for Future Research

The current study provides a comprehensive evaluation of risk and protective factors associated with sexting among Australian adolescents. The prevalence of sending sexts among study participants was 11.7%, which is lower than the recent figure of 19.3% reported by Mori et al. (2022). In the current study, sexts were operationalized as “sexually explicit images sent via their mobile phones”. According to Barrense-Dias et al. (2017), sexts may vary in the level of explicitness and modes of transmission. Therefore, narrowing sexting to sexually explicit images sent only via mobile phones may have resulted in lower rates of sexting. Future studies could implement sexting questionnaires where levels of sexual explicitness and various modes of transmission (through tablet and other electronic devices) are captured.

While this study provides valuable insight into sexting behaviors among young adolescents (mean age 14.5 years), it is based on cross-sectional data, which precludes causation. Therefore, it would be beneficial to examine the set of risk and protective factors in a longitudinal study to establish causal links between variables and ascertain which factors increase in importance (and significance) as young persons mature. Further, some measures used in the current study, such as interaction with prosocial peers and transitions and mobility, had low internal reliability but were retained as they were used in prior research (Toumbourou et al., 2019). These measures might not have emerged as significant predictors as they did not reliably assess the constructs of interest. Therefore, future studies could consider replacing these scales with others that have higher internal consistency.

Finally, future studies should also examine a range of risk and protective factors associated with the non-consensual sending of sexts (sext dissemination, sending sexts under pressure) and the sending of consensual sexts among heterosexual and non-exclusively heterosexual adolescents. Research conducted among a large sample of middle-school adolescents in the United States revealed that non-heterosexual orientation and LGBQ status was associated with nine times the odds of having sent a sext (Rice et al., 2014). Sexual and gender-diverse minority individuals are more likely to be stigmatized (Camp et al., 2020) and experience image-based abuse, whereby one’s sexual images are shared or posted online without the consent of the person depicted in such material (Henry et al., 2018). As such, the knowledge of risks and protective factors associated with non-consensual sexting and sexting among non-exclusively heterosexual young persons could inform sexting education and intervention measures specifically tailored for sexual and gender diverse minority youth.

## Conclusion

Current research on adolescent sexting is lacking a comprehensive theoretical approach examining a range of risk and protective factors associated with the consensual sending of sexts. It is also missing a systematic investigation into how the significance of these factors may vary across adolescent developmental stages. This study identified several risk and protective factors associated with adolescent sexting across individual, family, and community factors. These findings illustrate the usefulness of the Social Development Model in predicting the potentially risky behavior of sext sending among adolescents. This research also enhances understanding of when specific risk and protective factors become prominent across adolescent developmental stages. Sexting prevention and intervention measures could target a range of factors across individual (substance use, depressive symptoms, sensation seeking, adaptive coping) and social units (parental overcontrol, family conflict, substance availability in the community) simultaneously for maximum effect. These approaches, however, should be tailored to adolescent age as various risk and protective factors become more prominent across different developmental stages.
